# The New York Institute of Stomatology

**Published:** 1896-09

**Authors:** 

**Affiliations:** The New York Institute of Stomatology


### Reports of Society Meetings.

### THE NEW YORK INSTITUTE OF STOMATOLOGY.

A MEETING of the Institute was held at the residence of Dr. C. W. Meloney, No. 54 West Forty-eighth Street, New York, Tuesday evening, June 2, 1896, Dr. Benjamin Lord, President, in the chair.

The minutes of the previous meeting were read and approved.

Upon the call for communications from committees, Dr. Bogue, chairman of the Committee on Operative Dentistry, responded.

*Dr. E. A. Bogue.*—If the President will permit me, I would like to ask Dr. McNaughton to read his part of the report.

*Dr. S. H. McNaughton.*—The preparation with which I have had some success as an obtundent is composed of equal parts of cocaine and thymol, made by heating the two in a test-tube, the thymol being melted, and this in turn dissolved the cocaine; or it may be made by grinding the two together, a thick syrupy fluid being the result. For sensitive dentine, I use it by placing a piece of asbestos paper saturated with it in the cavity and over as broad a surface as possible, covering this with zinc phosphate, and allowing it to remain two or three days or more.

Harlan and others have shown that medicaments placed in the pulp-canal will traverse entirely through the dentine and cementum; that some substances do enter the living dentine is proved by the discoloration produced by amalgam fillings, by nicotine, by cannabis indica; it seems more than likely that arsenous acid is carried into the dentine by the living matter of the tooth. We often observe, just as the rubber dam is removed from teeth, that the teeth most thoroughly dried out by hot air have a lighter or whiter appearance; especially is this the case if an oil has been used to saturate the dentine. After a time the natural color returns. There has evidently been some change in the amount of moisture in the tooth, so that the theory of absorption of cocaine by dentine may not be a far-fetched one, especially as it seems to be conceded that by cataphoresis cocaine may be forced into the tissues.

Some cases have seemed to prove that the compound cocaine and thymol is really an obtundent. I shall only give two or three.

Case I.—Mrs. P.—Cavity in distal surface of superior bicuspid; began to prepare for filling, but finding it exceedingly sensitive, applied the cocaine mixture; she returned two or three days later, and there was almost no pain in thoroughly preparing the cavity. She said it was worth an extra trip from Yonkers.

Case II.—I had partially prepared a very sensitive cavity in the lateral incisor when a cavity was discovered in the central, and as my next patient had already come, I placed in the cavity in the lateral some of the cocaine and thymol covered with zinc-phosphate and made an appointment for three days later. At this sitting, when the preparation of the cavity in the lateral was completed, he asked if that was the same tooth that had been so sensitive the previous day, and, on being told that it was, said that nearly all tenderness had disappeared. He did not know of my using any obtundent.

Case III.—Young lady, aged twenty. I attempted to prepare approximal cavities in bicuspid and molar, but found both to be so extremely sensitive that I could do almost nothing. An application of the cocaine and thymol was made and covered with gutta-percha, and these were not again operated upon for over two weeks. The sensitiveness was somewhat lessened, and I was enabled to prepare both cavities to my satisfaction; but of all the cases I have tried where the preparation has been in two days or more, I consider this the nearest to failure. I think, perhaps, that the cocaine had leaked from under the filling, or that possibly enough had not been used, and perhaps too much time had elapsed.

I relate these cases believing the cocaine and thymol compound to be of assistance. The addition of potash to devitalizing mixture hastens its action, but it needs to be used with great caution.

Some of the other uses to which the cocaine and thymol may be put are to saturate pulp-canals, and to anæsthetize the tissue beyond the foramen preparatory to filling a root, to protect sensitive dentine while operating, as a vehicle for arsenous acid, etc.

*Dr. E. A. Bogue.*—Your Committee on Operative Dentistry begs to call attention to some of the recent utterances in regard to cataphoresis, both for local anæsthesia and for the bleaching of discolored teeth, and to give the results of a few experiments with the volt-selector, furnished by the Electro-Therapeutic Company.

It must be borne in mind that the most sensitive cavities with which we have to deal are those at or near the margin of the gum, or along the line of demarcation between the enamel and the dentine. When that line shall have been passed, the sensitiveness in any given tooth diminishes. The most difficult cavities to excavate and those most in need of obtundents are those between the teeth, and this is where cataphoresis is always difficult to apply, and most ineffective, if applied, so that it has been impossible for the writer to utilize it to advantage in this class of cases, excepting in readily accessible cavities in incisor teeth.

In those neglected cavities where, as Dr. Morton said, "the leathery substance at the bottom of the cavity can be peeled up, oftentimes exposing a sensitive zone," this mode of cataphoric obtunding is easily applicable. But the peeling up process (again according to Dr. Morton), even after the cocaine has been driven in electrically, gives so much pain that the writer wishes it might have been done before applying cataphoresis.

Neglectful patients are not generally the over-sensitive variety, but those who make immediate application for relief in case of any known defect in their teeth are the ones who are delicate and sensitive to the last degree, and who most need all the relief that can be procured. For that class of patients cataphoresis seems of little value.

It has another drawback that was strongly insisted upon by Dr. Morton at a recent Odontological Society meeting, and the writer cannot do better than to emphasize his warning. He said in substance, "It is astonishing how sensitive the nerves of a tooth are to even the slightest electric current; even one-half a volt is acutely felt, and a single cell of the Leclanché battery causes pain." This is quite true, and it limits the application of the instrument to a comparatively small number of cases, and these are the ones that need it least. The writer will enumerate few of the cases in which he used the volt-selector.

Mrs. T., a lady of perhaps fifty-five years of age; so intolerant of pain that when she first presented herself for treatment, five years ago, she would not allow an explorer to be put against the neck of the tooth, or into the cavities, without flinching so much as to render an accurate examination impossible. At that time the cavities in her teeth were obtunded with hot air first, and that was followed by pure carbolic acid and cocaine.

In certain proximate cavities where hot air could not be introduced, after drying the cavities as well as possible, carbolic acid and cocaine were first used, and afterwards zinc chloride in crystals, allowing these crystals to deliquesce in the cavity. This process has given excellent results in a larger number of cases than anything else that the writer is acquainted with, and these two methods have been pursued by him during the last twelve or fifteen years.

The lady whose case is now being described refers to the process as "the hot air treatment." A few days since a twenty-per-cent. solution of cocaine was applied with the electric current. The patient reported that the tooth was slightly sensitive to the operation, but it took about twenty minutes with a very disagreeable sensation to accomplish the same results that had always been accomplished for her by the "hot air process," as she called it. The second time that the selector was used, she declared very positively that her preference was for "hot air," and the applications that went with it. As this declaration was made when a cavity was but partially prepared, it was finished by using carbolic acid and cocaine followed by chloride of zinc, which accomplished the desired result, the tooth being excavated without further pain.

D. D., a brave little boy, ten years old; left upper molar; approximate cavity; exceedingly sensitive; necessary to fill where the temporary tooth, which had just fallen, had left space enough to work in.

Application of hot air gave pain for the moment; applied the cocaine solution (twenty per cent.) with the volt-selector, and the boy complained at every turn of the regulator, every increase of voltage, however minute; not that he was being hurt especially, but that the sensation was so disagreeable as to make him cry out.

After nine minutes of current, during which the indicator went up to "six," the current was discontinued; sensitiveness was diminished for a slight depth at the margin between dentine and enamel, but before all the decay had been removed the boy was bitterly complaining of being hurt.

Finished by applying chloride of zinc in crystals, which soon gave the ability to finish the excavation.

Mrs. E. said that the electric current was far worse than the pain of the tooth; that it was a very disagreeable feeling, and made her arm lame for a long time afterwards; she also said that, as a general thing, after a tooth was filled she heard nothing more about it, but in this case, the tooth operated upon ached for a day or so afterwards.

Mrs. J. said the first time that the writer used the electric current there was an alleviation of sensitiveness, and the work was much more easily performed. The second time she said she did not see much difference between that and the old way of doing, but was willing to have whichever way the operator said was better.

Cataphoresis has been successfully tried in four cases of bleaching,—three central incisors and one lateral. The lateral was considerably discolored with a bluish tint, arising evidently from extravasated blood in the pulp-chamber, which extravasation caused the bursting of the blood-corpuscles and the dissemination of the hæmoglobin into the dentinal tubes. The diagnosis of this condition was very clear after the tooth had been opened, because about one-third or one-half of the pulp was found alive, the rest dead. The other three teeth were yellowish, one of them having abscessed and having the pulp-chamber full of pus when the drill struck it; the other two teeth were yellow through the effects of a blow received many years previously, which had evidently destroyed both pulps. These three central incisors were all nicely bleached in from twenty to thirty minutes, but the lateral took an hour and required a voltage of "37" of the volt-selector, producing an amount of heat that was very perceptible to the operator as well as the patient. A professional friend, who assisted at these operations, gave it as his opinion that he had himself produced equally good results in bleaching by the use of hot air for drying the cavities first, and then pyrozone, without the electric current. Now the reason of this partial success is not far to seek. When the electric current is passing through a portion of the living organism, it naturally encounters different degrees of conductivity on the part of the tissues through which it passes. When the positive electrode is placed inside the cavity of a tooth, which tooth is composed of dentine somewhat analogous to ivory, enamel, still more a non-conductor, and cementum, a bony structure, it cannot be supposed that the current will follow any but the lines of least resistance, and it cannot be supposed that the same result can be obtained through a non-conducting substance that can be reached through nerve and muscle, or even through skin when moistened.

Dr. Morton, to whom the writer is indebted for his knowledge on this subject, says that he is profoundly ignorant of all strictly dental matters. Your committee would certainly not be so impolite as to contradict this assertion, especially in view of a little zone in one of the teeth which was bleached that for a long time resisted the bleaching agency. Why was this? It cannot, perhaps, be proved without more extended experiment, but it seems quite explicable upon the ground that the electric current was following out the line of least resistance, and so leaving certain dentinal tubuli that were off the direct line unaffected, until capillary attraction and osmosis eventually, after two or three days, did the work. Another local anæsthetic that does not seem to be very generally used among dentists, but nevertheless is of great value, is chloride of ethyl.

This substance has been more and more used in hospitals for the last six or seven years, in minor surgical operations, such as opening abscesses, amputation of fingers, and the removal of small growths, as well as for the extraction of teeth. Being put up in small glass tubes with brass caps over the orifice, no especial apparatus is required for its use. It evaporates so rapidly that the freezing-point is reached sometimes inside of two minutes, and anæsthesia generally in one minute if the surface is dry. What results may be reached in the near future with the help of the electric current we do not know, but up to the present the much-talked-of volt-selector must take its place as one of the many instrumentalities for local anæsthesia, and not pre-eminent for its safety or rapidity of action.

Dr. Brunton, of Leeds, recently sent the writer a little lamp in the wick of which is a coil of platinum wire to equalize the flame and prevent the lamp going out. A shaving an inch and a quarter long, cut from a platinum plate of No. 30 gauge, would answer very well, and could be attached to the wick of any lamp. It is rather efficient.

The attention of the chairman of this committee was called by Dr. Davenport to a method of banding teeth with gold, described in a paper by Dr. J. F. Adams and published in the *Digest* for January, 1896. Dr. Davenport seems to think that Dr. Adams's advice should be noticed by our members. The writer is pleased to say that eleven years ago a patient came into his office with a tooth banded in very much the same way by Dr. Allport, of Chicago, and that tooth was in good condition a few weeks ago. The writer has had two other teeth bleached since this paper was written, both of which have been bleached without the volt-selector, and so far as can be seen the results attained have been as good as with the volt-selector. It took, however, a little more time to do it. Dr. J. G. Palmer bleached two central incisors in the mouth of an English lady, who was written to, inquiring about them, and the answer to that letter stated that her teeth were gradually turning black, and she was very much disheartened. With Dr. Palmer's permission the patient was invited to the office of the writer, and it was found that a point not larger than the puncture that a small pin would make in paper, where the gold showed through the enamel, exhibited a dark spot; for the rest of it, the bleaching which Dr. Palmer did for her was admirable and was staying well.

*The President.*—The report of the Committee on Operative Dentistry is now before the meeting for discussion.

*Dr. Charles F. Allan.*—In the light of my personal experience the past three or four weeks, the report of Dr. Bogue and the remarks of Dr. McNaughton just made seem to me like ancient history. Cataphoresis has come to stay. The Wheeler volt-selector, upon which the doctor's remarks have been mainly predicted, I have never used and cannot criticise, but with the machine which is before us to-night I know we have a means of controlling the sensitiveness of dentine, and many other sensitive conditions of the oral cavity, which is simply perfect,—that is, absolute. We have only one idiosyncrasy to combat, and that is the peculiar, varying quality of the teeth of the individual to conduct electricity, but the result is sure. It is not only that in the end we have complete anæsthesia, but it is a fact also that the process itself gives no pain.

I think the main trouble in using the machine seen here (the Van Woert cataphoric machine) has been that too generally the operator uses all of the cells. I find that is ordinarily not best; generally half of the cells will do the work.

There has been with me the radical difference in making complete anæsthesia in some cases of from six minutes to forty-five minutes, the latter case being one to which I think I referred at our last meeting; and I think I said also at that time that I was quite certain that when I had that patient again in my chair the time would be very much abridged. This has proved to be the case; a very deep coronal and proximate cavity in a bicuspid tooth was completely anæsthetized in fifteen minutes. The average time taken by me has certainly not been over twenty minutes, and I

would expect in the future to have it cut down to twelve or fifteen. By using judgment in the number of cells used, and of the way in which the current is turned on, no pain need be caused,—that is, no pain that the patient will complain of.

But there is another phase of the subject. I think every operator using this process will want to be very careful in the selection of his cases,—that is, cataphoresis should only be used where there is an urgent necessity for it. Complete anæsthesia loses to us the diagnostic indication of pain, which is almost invaluable in the preparation of so many cavities. In many ways pain is the very best diagnostic sign we have, and we should go but slowly in depriving ourselves of its valuable help. In all of my cases so far I have used a twenty-five-per-cent. solution of cocaine.

*Dr. J. G. Palmer.*—I think I can answer Dr. Allan's remarks concerning pyrozone, and make a few remarks referring to Dr. Bogue's. Dr. Van Woert was right regarding the conducting properties of twenty-five-per-cent. solution of pyrozone, the ethereal solution; but Dr. Morton has a way of making that solution an aqueous solution, by evaporating it, and the electric current will pass through it, and will do the work of bleaching quicker. I am very much in doubt as to its doing it any better than the ethereal solution used with warm air, but it will do it a great deal sooner. In the case which Dr. Bogue alluded to, of the English lady, he omitted to mention one fact, that in this tooth which she thought was darkening somewhat, there was a gold filling, not removed at the time of bleaching. All metals should be removed from the tooth to have the best results. The other tooth had no filling in it whatever. The discoloration she discovered was just a line of this gold filling that had been allowed to remain in the tooth. In the use of the selector I had better results than Dr. Bogue has had, probably because I selected easier and more accessible cavities. I stood by Dr. Bogue's chair, however, observing some of the experiments he has narrated, and I must confess that, despite my own success with the apparatus, he succeeded better with cocaine followed by zinc chloride, as described. I think Dr. Allan is well within bounds when he says the average would be about fifteen minutes. I have never been able to produce complete anæsthesia in less than seven minutes; if I remember, the majority of cases have been much more than that, and I am probably more convinced than Dr. Bogue that the selector will produce satisfactory results, but I have not been able to see it in any case, as Dr. Allan has, without producing pain. I doubt if I can do it without producing some pain in the operation. I have not used this one which Dr. Allan has. I thought that the pain was due to the fact that I was trying to obtund the tooth in less time than fifteen minutes. In cases where I prolonged it to twenty minutes, turning up from fourteen to fifteen volts, there was decided success. Dr. Brown reported at a meeting in New Jersey that he had with a combination of fifteen per cent. cocaine with electrozone been able to produce complete anæsthesia in a minute and a half. In at least three cases of my own there was complaint that there was a great degree of soreness and irritation, like that which is consequent upon a prolonged operation and where the ligature has to be forced under the gum, running over two or three days.

*The President.*—All must feel this is a most interesting subject. Will Dr. Brown, of Elizabeth, give us his experience and views?

*Dr. G. Carleton Brown.*—While feeling the honor you have conferred upon me by asking me to give my experience in cataphoresis to the Institute, I feel that there is really little for me to say that will be of benefit, as the more experience I have the less I seem to know about the matter.

Some time ago I reported some cases that I considered interesting to the Central Dental Association of New Jersey, among them being one in which I secured complete insensibility in one minute and a half. As this case has been considerably talked about, I wish to state that there was no chance for a mistake, as my assistant always manages the instrument and keeps a careful record of the time consumed in all operations. The cavity was a very sensitive one, being situated in the anterior cervical surface of the left inferior cuspid, and extending slightly under the margin of the gum. A fifteen-per-cent. solution of cocaine in meditrina (electrozone) was used, being applied to the gum for one minute and to the tooth for one-half a minute. This was done to enable me to put on the clamp painlessly, which was accomplished; and, finding that the tooth did not respond to the pressure of the clamp, I decided to start excavating, and see how far the tooth was really obtunded. To my surprise, I was able to excavate the entire cavity without the slightest pain.

A few days after reporting this case I filled the corresponding cavity in the opposite tooth, and to secure the same anæsthesia I had to keep the current on for fifteen minutes. The conditions were apparently the same, and what caused the difference I am unable to say. But while we do meet with these occasional reverses, still the ratio of successful cases is increasing, as any one may prove by keeping a careful record of all cases, failures as well as successes.

In regard to the average time required to produce the necessary insensibility, I think that Dr. Allan's fifteen minutes is more than is necessary; in most cases we are all apt, I think, to keep the current on longer than is really required, and time can often be saved by making several short applications rather than one long one. There is a tendency which I think should be carefully guarded against,—the inclination to use cataphoresis in a great many cases where it is unnecessary.

*Dr. Bogue.*—I would like to ask Dr. Brown whether in that minute and a half cataphoric case, using cocaine and ether and his cavity being at the margin of the gum, he took into account the fact that cocaine acts centrifugally and not centripetally, and acts to the peripheral extent of the nerves and not towards the centre.

*Dr. Brown.*—That might perhaps explain it; but, as I stated in my remarks, I applied the current first to the gum and afterwards to the tooth. I would not expect to get any effect on the tooth if I applied it to the tooth and the gum at the same time, as the whole electric force would, I think, pass through the gum.

*The President.*—Are there any further remarks to be made on this subject?

*Dr. Milton F. Smith.*—May I inquire what solution Drs. Palmer and Allan used; was it the aqueous solution?

*Dr. Palmer.*—I used the twenty-five-per-cent. aqueous solution.

*Dr. Smith.*—Have either used the cocaine with tincture of opium? My friend, Dr. Jas. P. Holder, says his best results have been secured with the solution of cocaine with tincture of opium instead of water. He also reported to me two cases in which he removed two pulps, and, he thought, painlessly. I am quite interested in that part of cataphoresis; it seems to me if we can do that, we certainly have made great progress in getting rid of arsenic, which some of us unfortunately are obliged to use.

*The President.*—It gives me pleasure to state that Professor Dawbarn has consented to speak for a few minutes this evening upon the subject of malignant growths. Will he kindly favor us now?

*Dr. R. H. M. Dawbarn.*—This subject of malignant growths is too extended for thorough treatment in a few minutes, and I do not know just what points you want to cover. What little I know about the matter might better come in clinical demonstration than as a didactic lecture; this is more matter for a long, formal essay than for a five minutes talk. However, I have here a growth which illustrates a number of points which should be of practical value to all of us. This is an upper jaw taken from a young gentleman of this State. The case was sent to me by Dr. Howe of this Institute, and was a very interesting one in a number of ways. I think when you examine the growth you will say that its superficial manifestation would not have justified a diagnosis of any such degree of malignancy and size of tumor as actually existed; the antrum being entirely filled.

*The President.*—Dr. Dawbarn will be very glad to be questioned later on the matter that he proposes to bring before us.

*Dr. Dawbarn.*—This case presented the following history: About a year ago or a trifle less this young gentleman, aged twenty-five, had trouble with the second bicuspid of his upper jaw, which ached for a considerable time and ultimately was drawn by a dentist in the town where he lived, not far from New York. On close questioning, the patient remembered, when he finally came to me, a very interesting point,—namely, that he had noticed that the end of the fang of the extracted tooth was soft instead of being of the usual petrous character. The dentist had commented on this as being odd, but nothing was thought about the matter. The discomfort continued,—not suffering, simply a feeling of discomfort there. Because of that feeling of discomfort, and because of a slight fulness in the roof of the mouth that he had noticed in the last two or three months, he went to Dr. Howe, who felt sure there was a serious condition. I think he was of opinion that there was a tumor; and he sent him to me. I must say that when the young man presented himself to me there was little wrong to be observed. He was a rather full-faced individual, and such slight prominence above the alveolus as existed was not noticeable except on comparing the two sides. The roof of the mouth on that side was a trifle swollen in appearance. He had no cachexia, such as one frequently sees in the late stages of malignant disease. I made my diagnosis, as I have repeatedly done, partly by aid of a hypodermic needle. I ran the hypodermic needle into what should be solid bone, and it went straight through. I propose, if you will permit me, to demonstrate this with a pin. Now, it is self-evident to any of us that there should here be the densest kind of dense bone; but in this case the pin goes straight through, you see, and meets only about the resistance of ordinary cartilage; you see how little there is to show for it,—a little swelling here in the roof of the mouth and again above the alveolus, over the antrum of Highmore, just behind the canine fossa. Thus far I had not made the diagnosis more than a presumptive one, as this softening might have been due, for instance, to osteoporosis accompanying an inflammatory condition there; but if there had been such a degree of osteitis with softening there would probably have been a previous clinical evidence of it, such as suppuration, an ulcerated root, or some other evidence of inflammatory bone lesion; and there was nothing of that kind. Then, too, if it had been merely a superficial softening from chronic inflammation of bone over the antrum, when the needle was pushed in through this very thin shell of bone I should have been able to move it with perfect ease; but instead of entering an air-space it was being pushed for the whole length of the needle into a solid mass; so it was evident that there was a large tumor there. Now, as to the variety of tumor, that was another matter. It might be a sarcoma, of several varieties; it might be odontoma, one of some half a dozen kinds; it might be a fibroma, an enchondroma, etc. As to odontoma, one small point is worth observing,—namely, that this man had a complete set of teeth, with none missing, except the one recently extracted. Now, in odontoma one regularly expects to miss a tooth; and pretty commonly the growth has developed from this tooth-follicle as a starting-point.

In order to make sure of the nature of the tumor, I cut from it a small piece, say, a centimetre long and a half centimetre in width, and sent it to a prominent pathologist, Dr. Ely, the president of the Pathological Society. He said it was a small spindle-celled sarcoma, and of an unusually malignant type. It is probably an epulis in origin, springing from the socket of the tooth that was extracted, and involving and softening the fang of that tooth, a microscopic examination of which would have enabled an early diagnosis to be reached. The growth filled the antrum simply because this happened to be the direction of least resistance. Now, of course, it became a matter for prompt operation. I determined to give him every chance I could of not having a recurrence; therefore I not only excised the entire upper jaw, but also completely excised the external carotid arteries on both sides, first tying in each instance all of its eight branches. Thereby the region formerly occupied by the growth becomes comparatively starved in blood-supply; and the fact seems of value in preventing return of the malignant growth.

The patient did well, and is now attending to his law practice, wearing a prosthetic appliance made by a member of this Institute.

Let us now compare this case with a rather similar one, showing that you cannot make your diagnosis by softening only (though that is of great importance, and if a dentist meets with a case where he can pass a needle or pin into what should be hard bone, it is high time that a surgical consultation should be held). A dentist of a near-by city told that young gentleman that he didn't believe there was anything serious the matter, and advised him not to go to a surgeon. Softening alone is not sufficient to enable one to make a diagnosis of a malignant condition there. Another little history will be in order. Dr. R. M. Davenport, of this city, sent to me two or three weeks ago a young lady who for a long while had been troubled with an ulcerated tooth. Perhaps Dr. Davenport would like to give the history of that case.

*Dr. R. M. Davenport.*—The lady came to me about the last of January, troubled with a discharge from an opening over the right superior molar. The abscess discharged two or three times a day, and sometimes oftener. Although the fistulous opening was over the molar, direct entrance to the abscess was made through the second bicuspid, which had been syringed out for some time by a dentist in the country. He filled the tooth, but a recurrence of the discharge followed almost immediately. I removed the filling, syringing with hydrogen dioxide and dilute sulphuric acid, which came out through the fistulous opening, but the abscess did not seem to get any better. The last injection of sulphuric acid came out through the nose, showing unquestionably that the trouble had gone through into the antrum. I then took the patient to Dr. Dawbarn.

*Dr. Dawbarn.*—Then, Mr. President,—continuing the history of this case,—when I examined the young lady, I noticed, and she herself had observed, that the bone of the roof of the mouth and that over the antrum was distinctly softened, so much so that with the fingers on both spots a sense of fluctuation could be observed apparently transmitted by fluid in the antrum. It was obvious that chronic inflammatory change (osteoporosis) had led to this bone-softening. After the operation I found that a needle would as easily penetrate the bone as in the instance of the patient with sarcoma, just discussed.

It was not necessary to examine the antrum by electric light, prior to operation, to make sure whether or not it was involved, because in this case we were sure of it, Dr. Davenport having observed upon at least one occasion that fluid which he injected into the root of the tooth most involved escaped from the nose on the same side.

About ten days ago I operated upon her, Drs. Davenport and Williamson assisting, using ether anæsthesia. I removed by gouge and chisel all bone obviously compromised, not by softening, but by suppurative involvement. The tooth-root, which had apparently been the starting-point of all the trouble, was chiselled across (and at a later date Dr. Davenport filled what little was left of the root), and the false passage into the antrum was freely enough enlarged to permit the entrance of the little finger. The antrum was irrigated with a saturated solution of boric acid and drained.

The subsequent history was uneventful. Healing was rapid. The drainage-opening is now nearly closed, and is no longer needed. Even in these ten days' time there is distinct resumption of rigidity by the adjacent bone which had formerly been softened.[1]

*The President.*—We trust that Dr. Dawbarn will not feel hurried, if he has any further remarks to make; if not, he surely is willing to be questioned.

*Dr. Allan.*—With a tumor present, is there any symptom or group of symptoms that would enable a physician to say to a patient, "If you don't submit to this treatment you will have cancer?"

*Dr. Dawbarn.*—No; several kinds of benign growths are capable at any time of taking on malignant characteristics. A tumor is not a safe thing to have, though at the time a patient comes to you it is purely a benign one. It may remain so, or else from unknown causes may, in several varieties, develop into carcinoma or sarcoma.

*Dr. Allan.*—I know a gentleman, about fifty years of age, who has little points of growth in the side of the lower jaw. Some of the points may be as big as the end of my little finger, and from that down to the size of a pea; he has had these all the time he has been a patient of mine, some ten or twelve years; they are growing slowly. As long as they are quiet and not troublesome, may they not as well be left alone?

*Dr. Dawbarn.*—I think it would be wise to have one of them excised and examined by a pathologist. Probably they are simple

---

[1] At the time this goes to press—a few weeks later—the bone is absolutely as rigid as ever.

osteomata and benign; but it is safest to make sure that no doubtful elements exist.

*Dr. J. Morgan Howe.*—I would like, if possible, to emphasize what Dr. Dawbarn has said, by calling attention to the necessity of our being familiar with the signs of disease, or at least suspicious of unfamiliar conditions, in order that we may not fall into the error that the gentleman did who examined this young man's mouth just before I did. The patient had been under the observation of a dentist and a physician for nearly six months, the second superior bicuspid having been removed last November. It was thought at the time that it was the cause of the tumor; but the swelling, he said, did not subside, and although he went back several times to both dentist and physician, he was assured up to the last that there was nothing serious the matter. I would like to say why I thought there was a suspicious and alarming condition. In the first place, the socket of the extracted tooth had not absorbed in the least; there was no shrinkage, and the cavity from which the root came was filled with a new growth of soft tissue. Next, I could easily and quickly exclude, as the cause of the tumor, all influence of the remaining teeth. They seemed to be in a normal condition, although loose; two molars and one bicuspid—the second having been removed—yielded to firm pressure in every direction without being sore, and the yielding was the giving of the socket to pressure, not looseness of the teeth *in* the socket. The swelling simulated an abscess in appearance, but was different from an abscess to the touch; pressure with the finger evidenced no pus, and there was an india-rubber-like elasticity, a resiliency different from the feeling of an abscessed tooth. Every dentist should have been suspicious of tumor in that locality, or connected with the teeth, not caused by the teeth, for if the dentist cannot find a cause in the teeth for the trouble, he should always be suspicious. The patient said the tumor had been lanced, but was not certain that any pus came away. I inserted a very thin bistoury into the tumor in three places, not expecting to get any discharge of pus, but to test the feeling of the yielding bony tissue. The bone seemed quite decalcified, so that I pierced it without any difficulty. This was another remarkable condition. There was one symptom lacking which I would have expected to find in a case of malignant disease. This young man had no pain of any serious character, but as I had known of many cancerous growths progressing to a considerable extent without causing great pain, I did not think that excluded the probability of this being a malignant growth. I would like, while I am on my feet, to refer to a similar case that came under my observation several years ago. The young man had been under the observation of a prominent dentist in this city for some months, and also of a prominent surgeon, but they neither of them seemed to suspect there was anything serious the matter. The patient said they discussed over and over again whether they should take out one of his molar teeth or not. When I saw him, two molar teeth were loose, in a very similar way to the loosening in the case of Dr. Dawbarn's recent operation. I informed this patient that the case was serious, and it being in Dr. Garretson's lifetime, I persuaded him to go that afternoon to Philadelphia. He was operated upon the next day, and the results were similar to those in Dr. Dawbarn's case, in that it was necessary to remove the entire upper jaw of one side; the disease recurred in six months, and he died within a year.

*The President.*—Does Dr. R. M. Davenport know anything further of the history of his case?

*Dr. Davenport.*—I know very little: the first bicuspid was removed when the patient was about fourteen, and the pulp in the second bicuspid was devitalized and the tooth filled. About a year after it was filled this abscess formed,—about four years ago.

*Dr. Wendell C. Phillips.*—I have enjoyed listening to what Dr. Dawbarn has said this evening, having had considerable experience with malignant growths in the vicinity of the nose, and I am always interested in anything Dr. Dawbarn has to offer in connection with his work as a surgeon. I rise to ask two questions, and if the president will pardon a few suggestions I will be glad to offer them. Did Dr. Dawbarn make a careful examination of the nasal cavities as to whether the man had any polyps, and were there any eye symptoms? I have seen quite a number of cases of malignant growth of the superior maxillary bone, and have found that the insidious commencement of this especial form of malignant disease, which is quite different from what we ordinarily speak of as cancer, allows the disease to get very deep hold on the patient before any special notice has been taken of the growth itself. It seems that these cases of sarcoma rarely give any pain until the growth gets large enough to cause pressure on the nerve-trunks. A patient came to me and I found several polyps in the nose, and instead of its being polyp structure the microscopical report came back that it was a sarcoma. It was primarily a sarcoma of the antrum, but the growth had already extended into the other sinuses, which is very common for it to do, and there was breaking down of bony structures. Of course it was fatal. Dr. Dawbarn has very wisely called our attention to-night to the necessity of taking precaution against recurrence. I know no form of cancer so liable to recurrence as sarcoma. I know no form that is any more surely fatal than is sarcoma. I don't mean to say that they all die, but nearly all do. Dr. Dawbarn has made every effort in this case to prevent recurrence, and the operation has certainly been very thorough. I am glad to hear Dr. Dawbarn speak so well of transillumination, which bears out my own experience. I recently heard a laryngologist say of it that it was good enough for a plaything, but he had never been able to discover that it is of any benefit. Had he known where to look for the light or dark spot, and known better the manipulation of the electrical appliances, he would have a little less trouble in getting favorable results. It was my pleasure to read the first paper upon this subject of transillumination before a dental society, in 1890, and the paper was published in the *Dental Cosmos* for January, 1891. The real secret of transillumination is to compare the influence of the reflected light in the two eyes and underneath them. As a rule, in antrum disease you will find darkness under the eye of the affected side, whereas upon the other side there will be a bright reflex.

*The President.*—Unless some gentleman has some question to ask Dr. Dawbarn——

*Dr. Jackson.*—Will Dr. Dawbarn kindly tell us whether sarcoma originates from the periosteum or from some other tissue?

*Dr. Dawbarn.*—In answer to the gentleman who last spoke, I think there can be no question in this case, since the bicuspid root had softened, that this thing began in the tooth itself, or more likely in its socket. It has a rather restricted basis, and it is incorporated with the bone in the roof of the mouth, but has no real pedicle. I think, as to prognosis, that the patient has a fair prospect; for the reason that this is a small fusiform-cell sarcoma, and that particular variety of sarcoma may be encapsuled; and if you will notice the specimen before you, this has a distinct capsule; therefore, instead of leaving me in doubt as to when I had removed all that was abnormal, I had a positive guide. I feel fairly confident that I removed all the abnormal growth. Regarding Dr. Howe's point, as to the question of pain, I find that malignant growths are apt to be painful; and as to this growth one would expect, having seen the tumor, that it would be painful, being so large. *A priori* it would puzzle any one, as it did Dr. Howe and myself, that there should be no pain. Doubtless it was the fact of development within the antrum, where it was not subject to pressure, that prevented much suffering. It would be a very easy thing with the abundant space now here that I should cauterize it, should there be a recurrence, charring those diseased tissues, and doing it, if necessary, more than once. I think the results of the case will be quite good. Those cases in which recurrence is fatal are very apt to be ones in which the patients do not come back to the surgeon in time. As a mere prophecy, I have a great deal of hope that this man will entirely recover; he may have a recurrence, but even so, with prompt treatment I think the final result will be recovery.

*Dr. Howe.*—Mr. President, at the last meeting of the Institute, Dr. Van Woert made some remarks which seemed to me so incorrect and misleading that I should have replied to them at the time, but for the fact that the meeting was overcrowded with material, and I did not wish to take up the time that had been allotted to others. With your permission I will call attention to them now, and test the reliability of some of the statements. In regard to two substances, guaiacol and pyrozone,—five-per-cent. and twenty-five-per-cent. solutions,—he said they are "absolute non-conductors" of the electric current. I doubt it very much: I think there is no such thing as an absolute non-conductor; even those substances which are regarded as insulators have their resistivity recorded and tabulated; hard rubber and porcelain stand, I believe, at the head of the list as affording the greatest resistance; I do not know about liquids excepting water, its specific resistance is recorded. But on this point I wish to object to the use of the words "absolute non-conductor" as misleading. Then, speaking of bleaching with pyrozone, he said that "upon careful experiment you will find that pyrozone solutions, either five per cent. or twenty-five per cent., are absolute non-conductors, and the addition of the electric fluid does not hasten or facilitate the operation in the least." I have bleached teeth with a twenty-five-per-cent. solution of pyrozone by the aid of the electric current for over a year, and I have never had such good results without the electric current as I have had with it, and I believe from clinical experience that the electric current has helped very much. Dr. Van Woert said that pyrozone, both five- and twenty-five-per-cent. solutions, were "absolute non-conductors," and I understood that this was his reason for asserting it would not be cataphorically driven into the tooth. I will, with your permission, test it here for us all to see; it was proposed that I test it publicly, and I am very glad to do it. Now, gentlemen, Dr. Allan has poured some twenty-five-per-cent. pyrozone into a wide-mouthed bottle, and I have inserted two German-silver wires through the cork into that solution; they stand in the liquid on opposite sides of the vial; I will just touch the wires that are in the solution with the electrodes; there are five cells connected now. The wires now are in the solution of twenty-five-per-cent. pyrozone, and you see that one milliampère is recorded by the milliampère meter. If the wires were larger, or if they were nearer together, or if they were inserted farther into the liquid, or if more cells were connected, in either case it would record more,—more current would pass. Now, another statement that was made was, "the conductibility of plain water with a pressure of thirty-one and one-half volts is just one-tenth of a millimetre to the half-drachm,—that is to say, when thirty-one and one-half volts are running through half a drachm of water the milliampère needle will show one-tenth of a milliampère." Now I don't know what Dr. Van Woert meant by plain water; I find that distilled water offers much greater resistance than tap water does; the reason, of course, we may suppose is that the chlorides, nitrites, etc., dissolved in our ordinary croton increase its conductivity. Distilled water has a very high resistance, but Dr. Van Woert said *plain water*, and which of the two he meant I do not know, but he said there would be *so much current* running through a *certain amount* of water. That is, I think, misleading, because the quantity of water that surrounds the electrodes does not at all influence the passage of the current; it depends upon the size of the electrodes, the distance that they are apart, and the extent of surface that is immersed in the liquid. That is easily shown. We will take this bottle of distilled water that I have here and put this cork with these wires in its opposite sides into it. On touching the electrodes to the wires we have two milliampères recorded, as you can see, the current passing through the distilled water. Now I will put the wires in the cork into tap water, and you will see the difference between the distilled water and the tap water. Please note where the needle stands; as I wanted to show that the amount of current does not depend on the amount of water, I put them now into this glass of water; you see that they are in about ten times as much water as before; the needle stands just the same, because the wires stand in just the same relation and are inserted to the same depth. The two electrodes might be in the river, and the current would be the same that it is in this glass, if their relations were the same and it were the same water. The points I wanted to call attention to are the use of the words "absolute non-conductor," the positive statement that the current would not pass through the solutions of pyrozone, and the idea conveyed that the amount of liquid influenced the current. I might have shown as to the pyrozone that the three-per-cent. watery solution of pyrozone conducts about twice as well as distilled water, so that it appears that the peroxide of hydrogen increases the conductivity of water very much.

This is electrozone which Dr. Allan wants tested. It is a very good conductor; you will notice that the needle will get very much excited as soon as I touch the wires. We cut down the battery from thirty-one to five cells, and we still have more than the milameter can measure.

*Dr. G. S. Allan.*—That would explain the success Dr. Brown had using cocaine and ether in electrozone.

*Dr. Howe.*—If we are sure that conductibility is the thing to be desired.

*Dr. G. S. Allan.*—I think it probable that the conductibility of the bleaching agent employed will directly influence the results obtained.

*Dr. Howe.*—That remark of Dr. Allan's calls up to mind a thought that has come to me recently: that it may be we will find that the hydrochloride of eucaine, which is a new agent that has been tested abroad, and is made by Schering & Glatz, may be of service to us in producing anæsthesia of dentine; but I hope that electrozone may prove to be a help.

*Dr. G. S. Allan.*—And it would be necessary to use a weaker solution of cocaine in electrozone in order to obtain equal results.

*Dr. Howe.*—That may be, but still its conductive properties may be very different, and its action on dentine may be very different from what it is on soft tissue; all these things are matters for experiment rather than for arriving at conclusions by reasoning.

Adjourned.

S. E. Davenport, D.D.S., M.D.S.,
*Editor The New York Institute of Stomatology.*

